# Average and Local Effect of Thermal Fatigue on the Coefficients of the Thermal Expansion of a Complex Continuous Composite Fibre Used for Car Clutch Facing: A Multi-Technique Study

**DOI:** 10.3390/ma16175833

**Published:** 2023-08-25

**Authors:** Camille Flament, Bruno Berthel, Michelle Salvia, Olivier Graton, Isabelle Alix

**Affiliations:** 1 Laboratoire de Tribologie et Dynamique des Systèmes, UMR CNRS 5513, Ecole Centrale de Lyon, Université de Lyon, 36 Avenue Guy de Collongue, 69134 Ecully, Franceolivier.graton@ec-lyon.fr (O.G.); 2 Valeo Matériaux de Friction, Rue Barthélémy Thimonnier, 87020 Limoges, France; isabelle.alix@valeo.com

**Keywords:** composite material, thermal fatigue, polymer ageing, coefficients of thermal expansion, digital image correlation (DIC), acoustic emission (AE), DMA

## Abstract

The clutch facing is a complex organic matrix composite in dry clutch systems. When the clutch engages, there is a sliding contact between the clutch facing and the mating surfaces, resulting in temperature increases of up to 300 °C. These thermal cycles activate several mechanisms that can have consequences on such material: cracking and, more generally, the ageing of the polymer. The thermomechanical properties of the material therefore evolve according to the number of thermal cycles. This study focused on investigating the local and average evolution of the coefficients of thermal expansion (CTE) of clutch facing as a function of thermal cycles. Several techniques were employed, including image stereocorrelation for determining the CTE, Dynamic Mechanical Analysis (DMA) tests for monitoring the ageing of the material and acoustic emission for highlighting the damage. The results showed that the average CTE decreased as a function of the temperature and the number of loading cycles, while locally, it increased in some areas and decreased in others. These differences appear to be the result of material heterogeneity (actual yarn tracing, etc.) and interaction between cracking and ageing mechanisms in the polymer matrix. Indeed, thermal cycling led to cracking and additional crosslinking, which is influenced by ageing conditions.

## 1. Introduction

**Figure 1:**
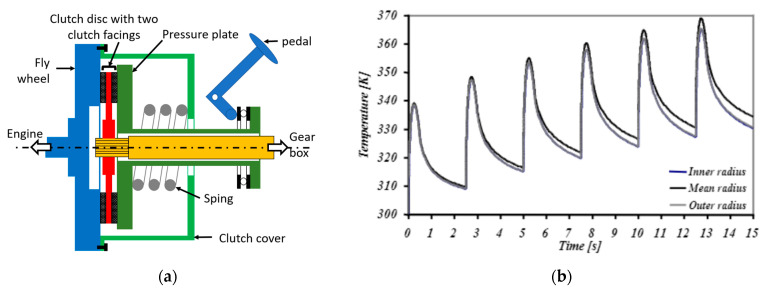
(**a**) Schematic diagram of a clutch; (**b**) simulated temperature variation of a clutch facing under successive engagements [4].

In vehicles, the dry clutch is used to connect or disconnect the gearbox from the engine, allowing the driver to change gears. In most cars with a manual gearbox, the dry clutch is located between the engine flywheel and the gearbox input shaft. A dry clutch operates by friction. When engaged, torque is transmitted by squeezing the clutch disc (with two clutch facings) between the engine flywheel and the pressure plate connected to the gearbox (Figure 1a). When the clutch pedal is depressed, the clutch is disengaged and the engine power is temporarily disconnected from the gearbox.

When dry clutch engages, there is a sliding contact between the clutch facing and mating surfaces, dissipating kinetic energy and generating heat. The temperature variation of the clutch facing can be divided into two stages [1,2,3]: a very fast rise in temperature, which can reach 250 or 300 °C in the case of repeated engagements for less than a second (Figure 1b), followed by cooling. A clutch facing is therefore subjected to different types of loads: tribological and thermomechanical stresses during clutch engagement (slipping with a temperature rise), followed by mechanical stresses (contact pressure and a centrifugal effect due to the speed of rotation at the engine output).

**Figure 2:**
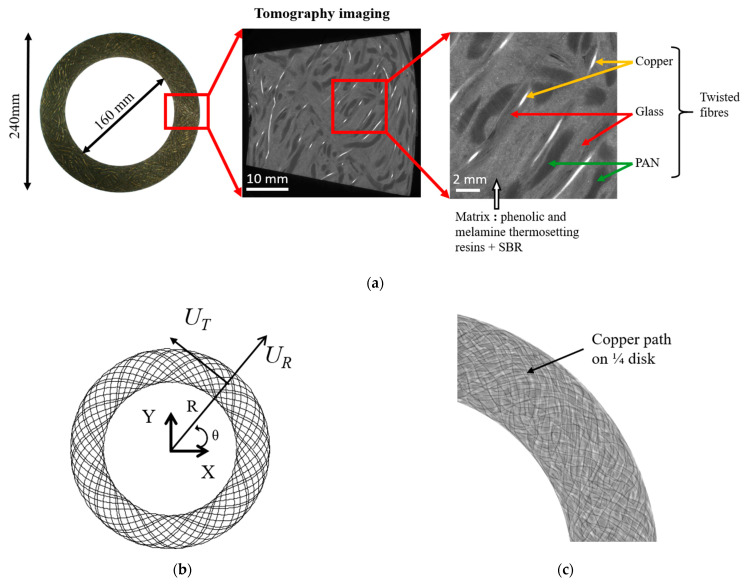
(**a**) Clutch facing and tomographic images of the composite material, (**b**) schematic preform and (**c**) X-ray tomographic view of a clutch facing with the copper path.

A clutch facing therefore needs to have a number of different properties to work effectively, including a high coefficient of friction, a good wear resistance, a high thermal stability, a low density and good mechanical properties. To achieve the best compromise, the materials used today are polymer matrix composites [5]. The matrix is made up of a mixture of several incompatible thermosetting polymers, generally toughened using rubber and various fillers. The reinforcements are in the form of yarns composed of different materials [5]. The choice of materials that make up the composite largely depends on the application (car, truck, racing, etc.), but in all cases, the matrix and the reinforcements play a role in the thermal, mechanical and tribological properties [3,5,6,7]. The orientation of the reinforcements must enable them to withstand stresses in all directions, and, due to the manufacturing process, the tracing is three-dimensional with variable angles of intersection [7,8] (Figure 2). All this makes it a material that can be considered complex in terms of the composition and organisation of the reinforcements.

The majority of studies on the behaviour of clutch facings have been numerical and focused on the engagement phase. This phase is critical, as it can generate temperatures and stresses higher than the material can withstand, ultimately leading to thermal failure. Numerous studies have been carried out to solve the problem of the thermoelastic effect in a sliding contact as a function of parameters such as the sliding speed and contact pressure [9,10,11,12,13,14,15] or to the transient response during the engagement phase [16,17,18,19]. In the latter case, the difficulty arises from the fact that the engagement is very short, so the system does not reach a stabilised state. Many authors have used these numerical methods to perform numerical simulations of clutch facings to determine the surface temperature [20,21,22,23,24], mechanical stress [5,25,26] and wear rate [27,28]. For instance, it has been observed that the highest temperature occurred at the midpoint of the sliding period, and its peak value was observed at the outer radius [29]. The sliding speed [1,30] or repeated engagements [4,31,32] have a significant effect on the thermal behaviour of dry friction clutches, and surface roughness can lead to very high localised temperatures [33]. In terms of materials, other studies have shown that a small clutch facing thickness has a significant effect on the temperature and contact pressure [34] or that it is possible to optimise the thermal properties of the clutch facing by the judicious choice of yarn tracing parameters [7].

Experimental studies focus mainly on the tribological behaviour of new friction materials [35,36,37,38,39] such as non-woven fabrics [40] or environmentally friendly friction materials [41], which have interesting properties. The tests are carried out on standard laboratory devices [6] or real tests in the automotive industry [3] and show, for example, the influence of the sliding speed on the coefficient of friction [42]. The mechanical or thermomechanical properties depend on the manufacturing process [43] and are generally obtained by measurements during tests performed on samples cut from clutch facings and on as-received materials [5]. Although the materials used for clutch facings are subjected to repeated thermal stresses, little research has been carried out on their evolution and damage under thermal fatigue loading [44]. As mentioned above, the clutch facing is made up of a continuous fibre composite material in the form of a ring with an organic matrix. The thermal fatigue of polymer matrix composites involves many phenomena that can modify the thermomechanical properties of these materials.

In the case of continuous fibre-reinforced polymer matrix composites, thermal fatigue can lead to matrix cracking, fibre/matrix debondings and delaminations, depending on the composite components and the orientation of reinforcements [45]. These damages are mainly due to the difference in the coefficient of thermal expansion (CTE) between the fibres and the resin. Physical (reversible) and chemical (irreversible) changes in the polymer matrix can occur when the composites are exposed to high temperatures. When exposed to a temperature just below the glass transition temperature (*T_g_*), a glassy polymer undergoes physical ageing, corresponding to a slow structural relaxation of the material that tends to converge towards a metastable state of thermodynamic equilibrium [46,47]. It is manifested by a gradual loss of free volume within the material, resulting in a restriction of molecular motions. From a mechanical properties point of view, this ageing process can be either favourable or detrimental to polymer applications. The material becomes stiffer and more resistant to creep [48,49] but shows a drop in ultimate strength and toughness [50]. These changes in properties are thermoreversible and can be removed after heating above *T_g_*.

Unlike physical ageing, thermal cycling can lead to irreversible modifications of polymers due to different chemical reactions that can take place at high temperatures, particularly in an oxidative environment (air). Thermosetting polymers consist of a set of covalent bonds (cross-links) connected across the polymer (three-dimensional network) formed during the curing process. Curing conditions (time, temperature) can lead to an incomplete cross-linking network in the matrix, notably due to the onset of vitrification, the transition from the rubbery to the glassy state [51] during polymerisation. Above vitrification, the cure mechanism is controlled by the molecular diffusivity and does not come to end. In the case of under-cured thermosetting resin, post-curing can occur. Such phenomena were observed in [52] on carbon-reinforced bismaleimide (BMI) resins subjected to thermal loading or in [53] on E-glass-reinforced epoxy subjected to self-heating associated with cyclic loading at a frequency of 30 Hz. Besides postcuring, which is mostly the dominant phenomenon, at the very early stages of exposure to thermal stress [51], thermosets may undergo degradation by oxidation as chain scissions and volatile releases. Lafarie-Frenot et al. prepared fully cured epoxy resins (DGEBA-DDS) (in order to avoid the occurrence of post-curing) which were subjected to thermal ageing (up to 5 days under 2 bars of oxygen at 150 °C) [54,55]. Using dynamic mechanical analysis (DMA), the authors pointed out a decrease in the main relaxation associated with the glass transition *T_g_* of the aged materials as compared to the neat resin, which was attributed to chain scissions. Wang et al. showed using FTIR and mass loss measurements that oxidation induces chain scissions in the bismaleimide (BMI) matrix of a T700 carbon fibre-reinforced BMI composite aged up to 1000 h at 200 °C and the removal of volatiles [56]. These phenomena (including post-curing, chain scissions and volatile releases) cause the shrinking of the matrix, which initiates the cracking of the composite matrix and the weakening of fibre/matrix interfaces, which adversely affects the use properties of composites.

The oxidation of composites occurs first on the surface and is controlled by the diffusion and reaction rate, which depend on the composite components and reinforcement architecture. Thermoset networks offer a wide range of thermal ageing performances, depending on the resin formulation and structure [51,57]. For example, Wang et al. have shown on phenolic networks that the higher the crosslinking rate, the higher the thermal oxidative stability, as the free volume, and therefore oxygen diffusivity, decreases [58]. Concerning the reinforcements, human-made engineering fibres such as carbon or glass have better thermal performance in air than lignocellulosic fibres. Carbon fibres are almost unaffected by temperatures in air below 400–500 °C [59,60]. E-glass fibres do not show any mass loss during high-temperature exposure but show strength degradation when exposed to temperatures above 350 °C due to the growth of surface defects [61]. When natural fibres are exposed to temperatures above 250 °C, they start degrading, mainly due to the presence of lignin [62,63]. The influence of the fibre orientation and stacking sequence on thermal ageing has been investigated by different authors [55,64,65,66,67,68]. All these authors have shown that the presence of fibres crossing the surface reduces the oxidation resistance of composites, as oxygen diffusion is higher along the fibres. In addition, microcracks in the matrix provide permeation pathways through which oxygen can penetrate the bulk of the composite [67,69,70,71].

In the case of isothermal thermal ageing, degradation is generally confined to the near-surface region of the composite (around 100 micrometres, depending on the polymers), whereas the propagation of matrix cracks from the surface to the bulk of the composite is accelerated by the coupling between oxidation and repeated thermal loading [72,73].

Disperse rubber particles in the matrix are often used to improve the toughness and fracture behaviour of thermosetting matrices by reducing their cracking susceptibility. The rubbers used are of several kinds: for example, carboxyl-terminated polybutadiene/acrylo-nitrile (CTBN), which gives the best results with epoxy resins [51,74] and diene elastomers such as styrene-butadiene rubber (SBR) or nitrile-butadiene rubber (NBR) [75], often used with phenolic-based systems, [76,77]. When subjected to heat under oxygen, even at medium temperatures (around 100 °C), rubbers can degrade. Sulphur-bonded (sulphur-vulcanised) rubbers have a higher mechanical strength than carbon-to-carbon bonded rubbers but are more susceptible to thermal ageing. Depending on the chemical composition of the rubber, chain scissions leading to the softening of the material, or a crosslinking reaction with the stiffening of the rubber, which becomes brittle, are observed [78]. In the case of diene rubber, hardening associated with crosslinking is observed in the case of NBR, while SBR undergoes a combination of chain scissions and crosslinking. However, the cross-linking mechanism predominates, so SBR hardens over time as NBR [79,80]. This effect may significantly reduce the influence of rubber particles on the toughness of rubber-thermoset blends with the ageing time.

The main objective of this study is to investigate how thermal cycling impacts the thermomechanical properties of automotive clutch facings made from a matrix based on a mixture of three components: melamine resin, phenolic resin and a diene rubber (styrene-butadiene rubber (SBR)). The inclusion of phenolic resin is related to its high chemical resistance, good heat resistance, dimensional stability and high mechanical strength. The melamine resin improves the mechanical, frictional and wear properties of phenolic-based friction materials [81]. The addition of SBR improves the flexibility and the toughness of the thermoset mixture [77,82].

In this study, the clutch facings were subjected to thermal fatigue tests in air at various temperature levels (200 °C, 250 °C and 300 °C) and for different numbers of temperature cycles, with in situ measurements of the coefficient of thermal expansion (CTE). Various experimental techniques are available for the measurement of thermal expansion, such as optical diffraction, dilatometers and strain gauges. However, these techniques require specific samples, and the manufacturing process of the studied composite makes it difficult to define an elementary representative volume. Therefore, an experimental setup was designed to perform tests on the whole specimen [8]. Digital Image Stereo Correlation (DISC) is one of the most popular full-field measurement techniques. It is a contactless optical experimental technique that measures full-field displacement with an accuracy of less than the size of a pixel. This technique is used to assess the mechanical and thermal properties of a wide range of materials, such as the coefficients of thermal expansion of polymers [83,84] or composites [8], the thermomechanical deformations of photovoltaic laminates cells [85] or the mechanical properties of composite materials [86,87]. This technique is therefore an interesting means of analysing local thermal behaviour. As the clutch facing alone was non-planar, since it was not held flat by the clutch system, it was necessary to use a stereo vision system, which allows for the study of non-planar specimens and out-of-plane displacement.

As previously mentioned, the thermomechanical behaviour of organic matrix composites can be modified by post-polymerisation reactions and resin oxidation under the influence of the thermal environment. After thermal fatigue cycles, various tests were carried out to characterise changes in the fatigued composite material. Dynamic mechanical analysis (DMA), which provides knowledge about the molecular motions of polymers, was used to obtain information on the chemical and mechanical changes associated with matrix ageing [52,55]. Information on the release of volatile products was obtained by measuring weight loss. The optical microscopy of the sample surface provided a detailed description of the cracking process [88]. Finally, tensile tests were performed on fatigued specimens using acoustic emission, which allows for the monitoring of damage within the material volume and provides a wide range of information on the latter. Indeed, when a composite material is subjected to an external load, very-high-frequency acoustic waves are emitted, and it is possible, for example, to correlate the amplitude of the acoustic emission signal with the damage mechanisms [89] to measure the extent of damage in composites [90] or to associate the number of acoustic events with the material damage mechanisms activated at different load levels [91].

Finally, the objectives of this study were twofold: measuring the effects of thermal cycles on the coefficients of the thermal expansion of a complex composite material used as a clutch facing and understanding the phenomena involved. Thermal cycling was performed in a climatic chamber specially adapted for this study, and the strain fields were measured using the DISC technique. At each measurement point, the coefficient of thermal expansion (CTE) was then defined as the local fractional increase in length per unit rise in temperature. To characterise the effects of ageing on the material, several complementary analyses were carried out: surface crack observation, weight loss, DMA and tensile tests coupled with acoustic emission.

The first part of the paper describes the clutch facing material, the experimental techniques and the procedures. The second part presents the CTE results, the complementary analysis of the material after ageing and discussions. Finally, the paper ends with a conclusion.

## 2. Materials and Methods

### 2.1. Clutch Facing Material

The studied organic clutch facing is an annular-shaped continuous fibre composite that transmits rotary motion from the engine to the gear box. It had an inner and outer diameter of 240 and 160 mm, respectively, and was 2.5 mm thick (Figure 2a).

The matrix is mainly composed of phenolic (Novolac, between 19 and 24 wt%) and melamine thermosetting resins (between 8 and 27 wt%) with sulphur-vulcanised styrene-butadiene rubber (SBR, between 20 and 34 wt%) [92]. The contribution of the phenolic and melamine resins as well as the SBR to the clutch facing properties has already been mentioned in the introduction. The matrix also contains carbon black (between 7 and 11 %wt) and barium sulphate (between 13 and 19%). Carbon black and barium sulphate are added to increase the heat resistance, wear resistance and coefficient of friction. Carbon black is also widely used for the reinforcement of rubber.

The reinforcement is a continuous yarn made of twisted E glass fibres (preferably one to three textured yarns from 600 to 5000 tex [92], depending on the model), polyacrylonitrile (PAN) fibres and copper filaments. The volume fraction of reinforcements is about 60%. Table 1 shows the mechanical properties of the constituent materials used in the composite. Reinforcing fibres influence tribological properties (friction and wear) [93]. PAN ensures good wear properties and a stable coefficient of friction, and copper has been found to enhance all major properties of friction [94].

Figure 2a,c show tomographic images of a clutch facing specimen. The images were acquired using X-ray tomography at the ESRF (European Synchrotron Radiation Facility, beamline: BM05) in Grenoble, France. The spatial resolution of the images was 12 × 12 × 12 μm^3^. For more details, please refer to [8].

The manufacturing process is well documented in [5,6,43]. Clutch facings are manufactured in the following steps (temperature and pressure data depend on the end use and geometry of the product):

- Impregnation: twisted fibres are impregnated with the resin mixture. Once impregnated, the yarns pass through an oven to release the solvents.
- Preforming: a weighed quantity of pre-impregnated wire is taken from the spool and fed to a vertically translating nozzle which projects the wire onto a rotating plate.
- Hot-pressing: the formed parts are placed in moulds, grooved to the required dimensions and then pressure-baked at a temperature of 190 °C for a few minutes.
- Curing: the material is overbaked up to two times at temperatures of 250 °C for at least 4 h.
- Machining: the clutch facings are ground, marked and drilled.

Ungrooved and undrilled specimens were studied to gain a better understanding of the behaviour of the structure. As mentioned above, during the preforming process, a machine uses a combination of uniform rotation and radial translation to guide the impregnated fibres. Each movement has a different frequency, resulting in a specific fibre arrangement illustrated in Figure 2b. This is described in Cartesian coordinates by:

(1) $$\left\{ \begin{array}{l} X\left(\theta \right)=\left[R_{0}+\frac{A_{0}}{2}\sin\left(\theta \times L_{b}\right)\right]\cos\left(\theta \right)\\ Y\left(\theta \right)=\left[R_{0}+\frac{A_{0}}{2}\sin\left(\theta \times L_{b}\right)\right]\sin\left(\theta \right) \end{array}\right.$$

where *R*_0_ is the Mean radius of the clutch facing, $A_{0}=\left(D_{out}-D_{in}\right)/2$ is the track width with *D_out_* and *D_in_* being the outer and inner diameters and *L_b_* is the preforming ratio, which is the number of sin waves per 2π phase angle.

The actual arrangement of the clutch facing’s fibres is generally more heterogeneous, as certain movements might take place during the preforming and curing steps, leading to alterations in the pattern of the impregnated yarn. The copper path can be seen in the tomographic image of a quarter of a specimen shown in Figure 2c. From X-ray tomography measurements, previous work [8] has shown that at the scale of the clutch facing, the actual organisation of the fibres was quite close to that obtained from Equation (1). The differences were more localised. The plot shown in Figure 2b can therefore be used as a first step towards understanding the behaviour of the material. This fibre organisation allows for assuming that the behaviour of the composite material used for the clutch facing is orthotropic.

**Figure 3:**
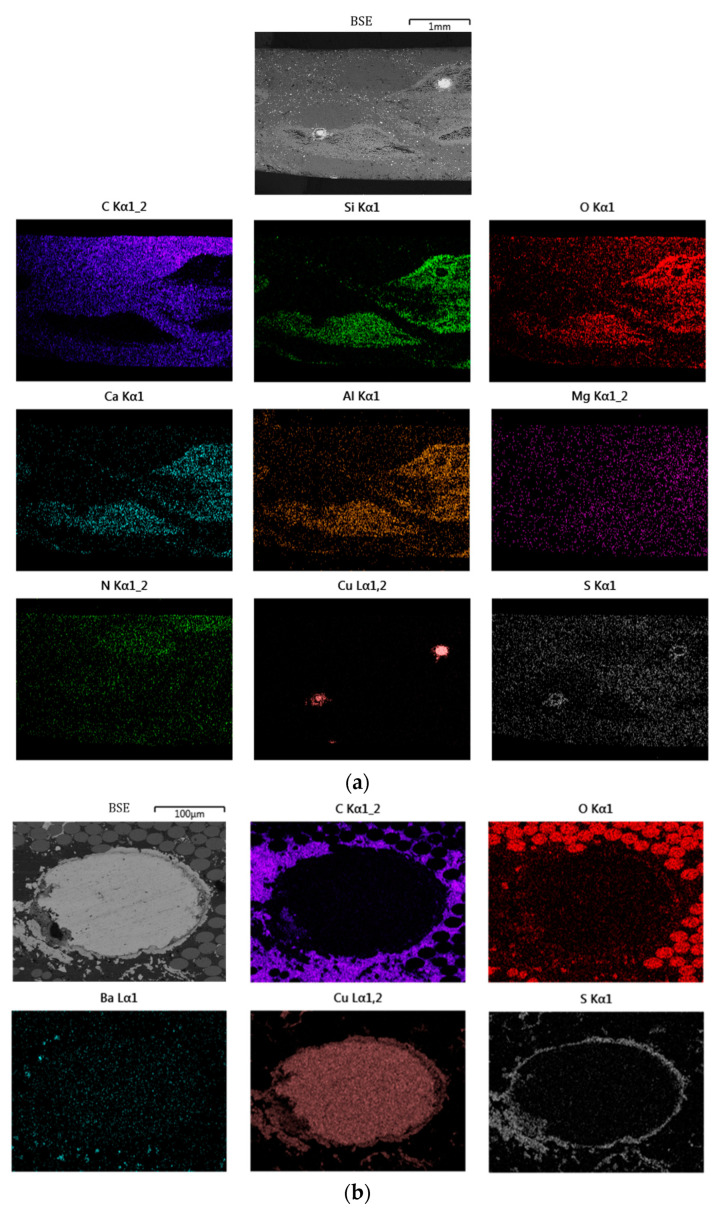
(**a**) SEM picture performed on a clutch facing radial cross-section in BSE mode (BSE) and corresponding EDS elemental maps of carbon (C), silicon (Si), oxygen (O), aluminium (Al), calcium (Ca), magnesium (Mg), nitrogen (N), copper (Cu) and sulphur (S); (**b**) magnified view around the cross-section of a copper wire I bSE mode (BSE) and corresponding EDS elemental maps of carbon (C), oxygen (O), Barium (Ba), copper (Cu) and sulphur (S).

Microstructural analysis was carried out using scanning electron microscopy (SEM) (TESCA MIRA 3, TESCAN FRANCE, Fuveau, France) equipped with energy dispersive spectroscopy (EDS) (OXFORD Instruments SAS, Les Ulis, France) on tangential cross-sections to gain access to the micromorphology of the composites. Prior to SEM analysis, a thin layer of gold is deposited on the composite cross-section to prevent sample charging. The EDS technique is used to obtain elemental distribution maps. The SEM image of the radial section of the composite in backscattered electron detection (BSE) mode and the corresponding EDS analyses are shown in Figure 3a. The BSE scanning mode can be used to distinguish areas of different chemical compositions, particularly if the atomic numbers are sufficiently distinct. Combined with energy dispersive X-ray spectroscopy (EDS), it is possible to obtain compositional maps of the chemical elements present in a material [95,96]. Carbon (C) is uniformly present in the organic matrix and PAN fibres. Oxygen (O), silicon (Si), aluminium (Al), calcium (Ca) and magnesium (Mg) maps indicate the location of glass fibres, which consist mainly of silicon, aluminium, calcium and magnesium oxides. Nitrogen (N) shows the PAN fibre position. The copper (Cu) map shows the location of copper filaments. The sulphur (S) distribution is fairly homogeneous in the matrix region except around the copper filaments, where higher sulphur concentrations are observed (higher colour intensity). 

These observations show (i) a good distribution of SBR particles (sulphur cross-linking bridges present in the elastomer) in the thermoset phases and (ii) that it can be assumed that a Cu–sulphide interphase structure was created between the copper filaments and the rubber [97]. This phenomenon is highlighted by the magnified view around the cross-section of a copper wire (Figure 3b). This picture also shows the composition of the yarn made of E-glass fibres (O), PAN fibres (N) and copper filaments (Cu) and the presence of the barium sulphate particles (Ba) and (S) as well.

### 2.2. Experimental Set-Up and Post-Processing for Measuring CTE Fields Evolutions

#### 2.2.1. Experimental Set-Up

**Figure 4:**
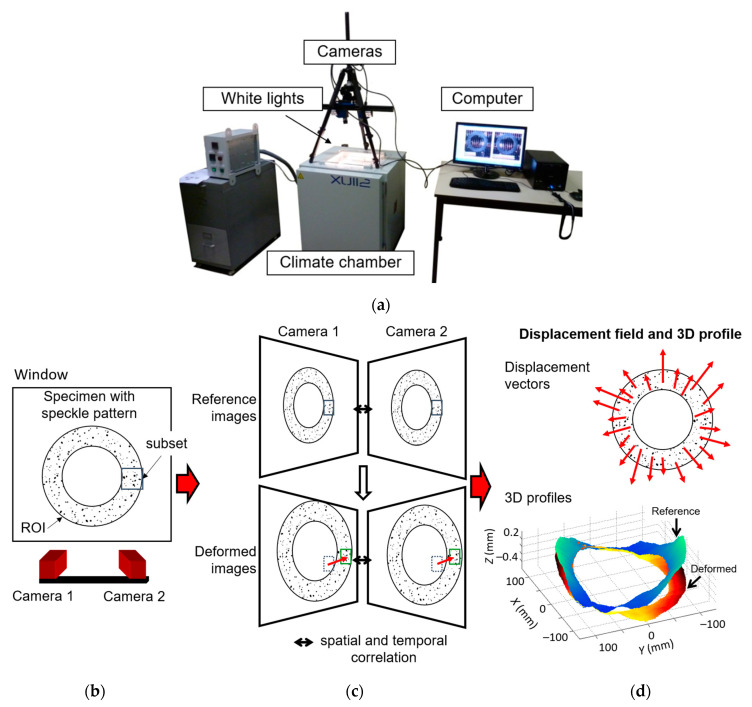
(**a**) Photo of the experimental set-up and principle of the Digital Image Stereo-Correlation (DISC) technique: (**b**) definition of the ROI, (**c**) subset tracking and (**d**) measured displacement field and shape of the deformed surface.

Thermal tests were performed using a XU112 climatic chamber specially adapted by the manufacturer France Etuves (Chelles, France). To determine the actual temperature, a thermocouple was placed on the surface of the disc. Before the tests, a calibration procedure was carried out by comparing the core and surface temperatures of the clutch facing. The specimen was placed horizontally in the climatic chamber without any restraint. The composite clutch facing samples were not perfectly flat, exhibiting wave amplitudes of up to 1 mm. The strain fields were therefore measured using the Digital Image Stereo-Correlation (DISC) technique, an optical method that provides displacement fields and 3D shapes of deformed surfaces (Figure 4d). Figure 4a shows a photo of the experimental setup, highlighting the climatic chamber with a window at the top for optical access to the specimen.

The DISC technique uses pairs of simultaneous images captured at different camera angles to determine 3D displacement fields on non-planar surfaces. Correlation only works with randomly textured surfaces. To achieve a sufficiently contrasting speckle pattern, the sample was sprayed in white and black. The principle of image correlation is as follows. On the surface of the sample, a specific region of interest (ROI) is selected and further sub-divided into smaller regions or subsets (Figure 4b). The DIC algorithm tracks these subsets to determine the corresponding reference subset positions in the deformed image (Figure 4c). The algorithm analyses the grey level of individual pixels within each subset to compare the deformed and reference subset. Optimised criteria for pattern matching have been formulated, and in this study, the Zero Normalised Sum of Square Difference correlation criterion was chosen [98]. Once the subsets are matched, the displacement vector of the centre of each subset between the reference image and the deformed image can be determined (Figure 4d). DISC combines temporal and stereoscopic matching.

To establish a correlation between stereoscopic images (Figure 4c), the correlation algorithm relies on information about the orientation and position of the cameras but also on their intrinsic parameters (distortions induced by the lens, etc.). Calibrating the stereo vision setup with specific pattern target images provides these parameters. The DISC method therefore makes it possible to distinguish between in-plane and out-of-plane displacements while providing access to 3D profiles (Figure 4d). The software used in this study is Vic 3D v7, developed by Correlated Solutions (Irmo, SC, USA).

The imaging setup consisted of two CCD cameras (AVT Pike F-421B) with a resolution of 2048 × 2048 pixels, producing monochromatic images with a 14-bit dynamic range. These 14-bit images were converted to 8-bit by the software. The cameras had a field of view of 250 × 250 mm^2^, resulting in each pixel on the CCD sensor corresponding to a square of 0.12 × 0.12 mm^2^ on the specimen. During calibration, the algorithm took into account the distortion present in the optical path [99]. High-temperature measurements can be affected by distortion caused by the climate chamber window and changes in the refractive index of the heated air [100]. To mitigate the effects of these two sources of error, the stereo system was calibrated through the window, allowing the additional distortion to be properly accounted for.

**Figure 5:**
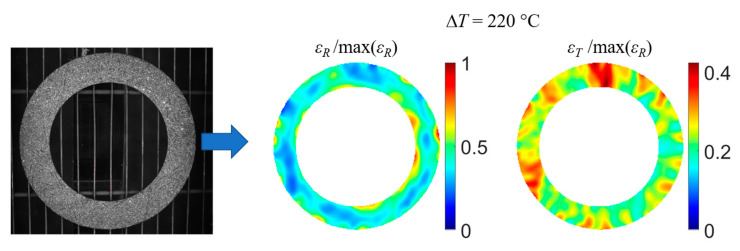
Radial and tangential strain fields (ε_R_ and ε_T_) measured for Δ*T* = 220 °C (*T* = 250 °C).

For this study, the size of the subset was 31 × 31 pixels, resulting in a spatial resolution of the displacement of 3.7 mm. The step size was seven pixels (distance between two points where displacements are measured). With these parameters, the device is equivalent to 1570 strain gauges. The Lagrange strain tensor was computed using the VIC 3D v7 DISC software. Strains were then obtained from the filtered displacement field using a filter box size of 15 calculated points. To reduce the effect of noise, several images were taken in a stabilised state at each temperature, and the strain tensors were calculated using averaged images. The strain error is temperature-dependent, and the maximum value was estimated to be 0.4 × 10^−3^ at 300 °C [8]. The normed radial and tangential strain fields (ε_R_ and ε_T_) for a thermal loading Δ*T* = 220 °C are shown in Figure 5 (Δ*T* = *T* − *T*_0_ with *T*_0_ = 30 °C).

#### 2.2.2. Coefficients of Thermal Expansion

The expansion of a material in response to a change in temperature is described by its coefficient of thermal expansion (CTE). It is generally defined as the fractional length increase per unit temperature increase (known as the linear CTE). For small deformations, the fractional length increase corresponds to the strain tensor (*ε*), and the coefficient of the thermal expansion tensor can be expressed as follows:

(2) $$\underline{\underline{\varepsilon }}=\underline{\underline{\alpha }}\;\Delta T$$

where *ε* is the strain tensor, Δ*T* is the temperature variation and α is the coefficient of the thermal expansion tensor.

Over a limited temperature range, the thermal expansion as a function of temperature is defined in its linear form, and the CTE is then constant. For certain materials and wider temperature ranges, it is temperature-dependent. In this study, the temperature range was 30 °C to 300 °C. Previous investigations have confirmed the orthotropic behaviour of the annular clutch facing and the fact that thermal strains increase linearly with temperature variation [8]. Finally, the equation below was used to determine the coefficients of thermal expansion in this study:

(3) $$\left[\begin{array}{ccc} CTE_{R} & 0 & 0\\ 0 & CTE_{T} & 0\\ 0 & 0 & CTE_{z} \end{array}\right]=\frac{1}{\Delta T}\left[\begin{array}{ccc} \varepsilon_{R} & 0 & 0\\ 0 & \varepsilon_{T} & 0\\ 0 & 0 & \varepsilon_{z} \end{array}\right]$$

where *ε_R_*, *ε_T_* and *ε_z_* (respectively, *CTE_R_*, *CTE_T_* and *CTE_z_*) are the tangential, radial and *z* components of the strain (coefficient of thermal expansion); see Figure 2b.

In this work, only the radial and tangential components of the coefficient of the thermal expansion tensor were calculated.

#### 2.2.3. Experimental Procedure and Post-Processing for CTE Measurements

**Figure 6:**
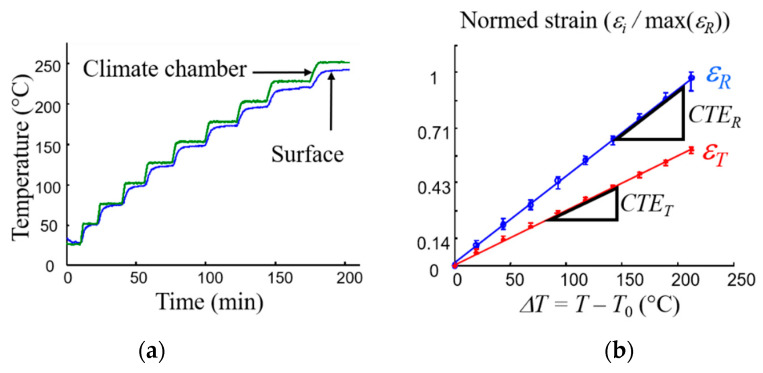

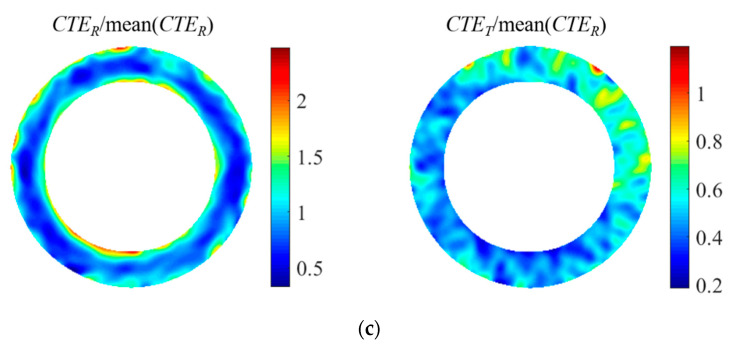
(**a**) Measured temperature versus time; (**b**) Normed strain versus ΔT (five identical clutch facings); (**c**) Example of fields of normed *CTE_R_* and *CTE_T._*

In order to measure the strain due to free thermal expansion, a reference state of the specimen was defined by taking image pairs at room temperature. The temperature was then gradually increased, and image pairs were taken every 25 °C from 50 °C to 300 °C, when the temperature of the specimen had stabilised (Figure 6a).

For noise reduction, twelve pairs of images were taken at each stabilisation temperature step, and the strain field was calculated from the averaged images. The strain resolution varied from 0.01% to 0.05% depending on the temperature [8,101]. Finally, Equation (2) was used to determine the coefficients of thermal expansion from the strain fields calculated at each temperature. The experimental device was validated with known materials (pure aluminium, aluminium oxide and unidirectional carbon-fibre-reinforced bismaleimide) before being applied to the clutch facing [8]. Figure 6b shows examples of the evolution of the mean radial (*ε_R_*) and tangential (*ε_T_*) strain as a function of the temperature. In the 30–250 °C temperature range, the composite showed linear behaviour, and two CTE were then calculated (*CTE_R_* ≈ 5 × 10^−5^ K^−1^ and *CTE_T_* ≈ *CTE_R_*/2). The experimental setup provides access to strain fields versus temperature, and CTE fields can then be identified. Examples of normed *CTE_R_* and *CTE_T_* maps for an as-received specimen are shown in Figure 6c. As shown in [8]:

- The *CTE_R_* essentially depends on the radius *R* (Figure 2b), being maximum at the ends of the track and minimum in the centre,
- The *CTE_T_* is close to an average value, despite some inhomogeneities due to material heterogeneity, which will be discussed later.

The purpose of this study was not to validate this method of measuring CTE fields or to explain their distribution (local organisation of the fibres). Readers interested in these topics should read [8]. The aim here was to follow the average and local evolution of the CTE as a function of the number of thermal cycles in order to obtain important information about the evolution of the material.

#### 2.2.4. Thermal Cycling Procedure

**Figure 7:**
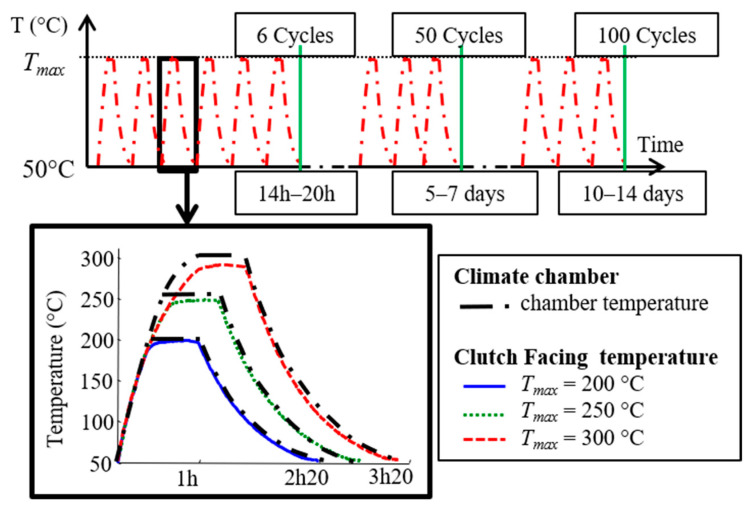
Thermal cycles.

To study the effects of thermal fatigue on the coefficients of thermal expansion of the composite, the specimens were subjected to thermal cycling at a constant maximal value (*T_max_*), as shown in Figure 7. Three maximum temperature values were chosen: 200 °C, 250 °C and 300 °C. For each of these three temperatures, the coefficients of thermal expansion were measured after 6, 25, 50, 75 and 100 cycles. The time taken for the temperature to rise and the duration of the plateau were chosen in order to stabilise the temperature within the material (very insulating material).

A homogeneous temperature in the volume was chosen mainly to study the ageing and damage of the material in the volume, with the CTE measurement also allowing for the in situ measurement of the evolution of the material on the entire clutch facing (shape/behaviour interaction). In the real application, the clutch lining is subjected to temperature gradients, but performing such tests makes in situ measurements and the analysis of the results more difficult. The next step would be to model the evolution of the material based on these homogeneous tests and then compare the numerical results with the temperature gradient tests.

### 2.3. Material and Damage Characterisations

This section describes the techniques that were used to quantify the effects of thermal ageing on clutch facing materials (damage and matrix changes).

#### 2.3.1. Surface Crack Measurement and Weight Loss

The cracking process was studied by observing the surfaces of the specimens. As the clutch facings were painted for DIC measurements, cracks were observed on specific specimens cut from a reference clutch facing and polished by metallographic methods before being tested. They were placed in the same climatic chamber as the entire clutch facing in order to be subjected to the same thermal cycles. After each test, an area of 12 × 12 mm^2^ was observed using a binocular microscope with an optical zoom from 0.67 to 4.5 (Olympus SZ61, Tokyo, Japan). These measurements provide a trend rather than a quantitative measurement. Weight loss measurements were also carried out on the same specimen using a precision balance with a resolution of 0.01 mg (SHIMADZU AUW220D, Kyoto, Japan).

#### 2.3.2. Dynamic Mechanical Analysis

Dynamical mechanical analysis (DMA) tests were carried out to highlight the eventual changes in the material, according to the thermal cycling. DMA is a convenient and sensitive technique for measuring the thermomechanical properties of polymers or reinforced polymers as a function of frequency and temperature. During a DMA test, a low-amplitude sinusoidal force is applied to a material and the displacement is measured, or a displacement is applied and the force is measured. Due to the viscoelastic properties of polymers, the displacement lags behind the force by a phase angle *δ* [102]. In the case of a tensile/compressive load on a specimen of known dimensions, it is then possible to determine the complex modulus E^*^ and the loss or damping factor tan *δ* of the material:

(4) $$E^{*}=E^{\prime }+iE^{\prime \prime }$$

(5) $$\tan\delta =\frac{E^{\prime \prime }}{E^{\prime }}$$

where *E*′ is the storage modulus and *E*″ is the loss modulus.

**Figure 8:**
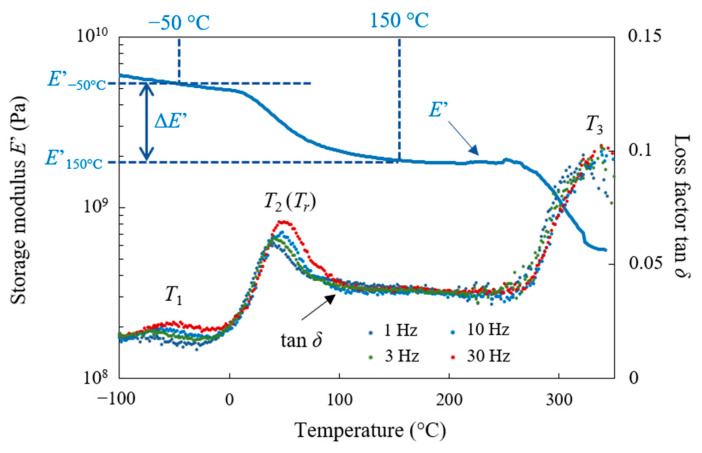
Storage modulus at 1 Hz and loss factor (1, 3, 10 and 30 Hz) of the as-received material vs. temperature.

**Figure 9:**
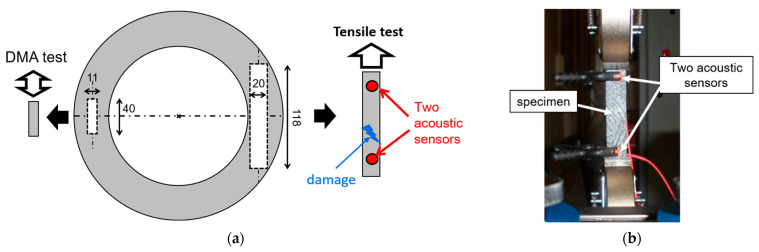
Principle (**a**) and picture of the tensile test (**b**).

This method is especially well suited for analysing the relaxation processes ((main or α) and sub-*T_g_*) of polymer-based materials [103,104,105]. DMA tests were carried out using a DMA50 of 01 dB METRAVIB (Limonest, France) on rectangular specimens cut into the clutch facing (size 40 × 12 × 2.5 mm^3^; see Figure 9a). Measurements were performed in the tension–compression mode with controlled dynamic displacement (±5 μm) in the linear viscoelasticity domain within a temperature range of −100 °C up to 350 °C at a heating rate of 1 K/min. The measuring frequency was from 1 Hz to 30 Hz. A typical plot of the storage modulus *E*′ and the loss factor tan *δ* versus temperature and frequency for the as-received material is shown in Figure 8.

Under thermomechanical loading, the as-received behaviour of the material in the temperature range studied is characterised by three frequency-dependent transitions, each with a peak in the tan *δ* spectra and a concomitant drop in the storage modulus. In ascending order of temperature and by considering the frequency of 1 Hz, a small peak is observed, centred around −68 °C (*T*_1_) and associated with a slight drop in *E*′ (Δ*E*′ ≈ 170 MPa), followed by another maximum at 36 °C (*T*_2_) with a sharp decrease in *E*′ by about 1000 MPa, and the last peak occurs around 320 °C (*T*_3_) with a decrease in E′ by a factor of 4.

The relaxation centred around −68 °C (*T*_1_) is probably related to local motions in the phenolic resin (micro-motions of the phenolic rings around the methylene bridges). Assuming, as a first approximation, that the relaxation phenomenon is governed by Arrhenius’ law (small frequency range (≈2.5 decades)), the activation energy was determined [106]. The low activation energy found (≈70 kJ/mol) seems to confirm the hypothesis that this relaxation was associated with the β-relaxation of the phenolic resin, which is weaker and more spread out in temperature than the main relaxation associated with the glass transition. The matrix also contained an elastomer, styrene-butadiene rubber (SBR). SBR is a non-polar elastomer. As phenolic (PF) and melamine (MF) resins are highly polar due to the presence of hydroxyl (OH) groups, the elastomer phase is dispersed in thermosets (see EDS analysis in Section 2.1) (incompatible mixture). The glass transition of SBR is around −40 °C [76]. There is no obvious peak associated with SBR in the DMA results. This may be explained by the partial solubility of phenolic resin in SBR observed by various authors [76,77]. The phenolic resin may participate in the SBR cross-linking network by the cross-linking of rubber chains via methylene bridges in addition to sulphur cross-links. This leads to a dense network with limited motion of the rubber segments between junctions and, consequently, a low amplitude of tan *δ*. This may be supported by the fact that the β-relaxation of the phenolic resin appears to be quite broad, which could mean the overlap of several contributions.

The *T*_3_ peak is quite wide, which could indicate the superposition of different contributions. It is probably associated with glass transitions, i.e., long-range molecular movements of phenolic and melamine resins (main relaxations). The shape of the loss factor varies according to the frequency tested. At low frequencies (1 Hz), the peak appears to be split. The probable reason is that the activation energies of the phenomena associated with each resin are different.

The activation energy of the *T*_2_ relaxation is approximately 230 kJ/mol, suggesting a large-scale cooperative motion of the main polymer chains. This relaxation could be related to the possible compatibility between phenolic resin and SBR, as mentioned above. Some authors [107] have highlighted a single tan *δ* peak at 40 °C on the DMA scan as a function of temperature on certain Novolac phenolic resin/NBR (nitrile-butadiene rubber) blends and attribute this result to the compatibility between NBR and phenolic resin. In the matrix of this study, the elastomeric phase is dispersed in the thermosets, and the tan *δ* peaks associated with thermosets are observed above 300 °C, but it is possible that compatibility between the rubber and thermoset may occur in some domains at the interface rubber/thermoset resin [77], resulting in the tan *δ* peak at *T*_2_, even though the compatibility of SBR with phenolic resin is a priori weaker than that of NBR, which is slightly polar.

This relaxation, as will be shown below, is highly dependent on material ageing and will be used as a marker for the latter. It will hereinafter be referred to as *T_r_* at 1 Hz. In fact, the *T*_1_ peak is not affected by thermal cycling, and some degradation may occur during the DMA test in the *T*_3_ temperature range.

In order to monitor the effect of ageing, several material properties (Figure 8) were extracted from the DMA sweeps at 1 Hz for each fatigue state (number of cycles and load temperature), and the results are presented in Section 3.2.2:

- the relaxation temperature: *T_r_*
- the storage moduli at fixed temperatures below and above *T_r_* (−50 °C and −150 °C): *E*′_−50°C_ and *E*′_150°C_
- the storage modulus difference: Δ*E*′= *E*′_−50°C_ − *E*′_150°C_

#### 2.3.3. Damage Measurements with Acoustic Emission

Tensile tests coupled with acoustic emission (AE) measurements were carried out on as-received and fatigued materials to obtain information on the damage in the volume of the material that had undergone different thermal cycles [108] (Figure 9).

When damage occurs or propagates in a material under stress, some elastic energy is released, which propagates through the material. This phenomenon is known as Acoustic Emission (AE). In composites, AE signals are generated in bursts associated with individual emission events occurring within the material such as matrix cracking, delamination or fibre failure. The signals are picked up by piezoelectric sensors coupled directly to the surface of the part [109,110]. AE behaviour can be characterised by the cumulative number of AE signals or events as a function of time and/or by analysing the parameters associated with the hit signature associated with the onset or growth of damage [111].

AE testing does not detect pre-existing defects in the material but rather their growth as they occur, the creation of new defects and their propagation. The Acoustic Emission (AE) monitoring is therefore an effective technique for investigating the behaviour of materials under stress in relation to their initial damage state. Specimens were then cut into the clutch facing in the tangential direction (size 118 × 20 × 1.8 mm^3^, Figure 9a) and subjected to tensile loading performed at an imposed displacement rate of 1 mm/min until the complete failure of the specimen. The load, the strain field and the AE signals were recorded during the test.

The testing machine used was an INSTRON model 3345 (Norwood, MA, USA) with a maximum capacity of 5 kN. Digital image stereo correlation (DISC) was used to determine the strain field (Section 2.2.1).

Acoustic emission (AE) signals were acquired and analysed using a Mistras AEwin system from Physical Acoustics Corporation (Princeton Junction, NJ, USA). Two piezoelectric sensors (type R15, Physical Acoustic Corporation) were used to detect the signals during the tests. These sensors were firmly attached to the sample using silicone grease and clips. The two sensors were positioned 70 mm apart at opposite ends of the specimen (Figure 9b). The threshold of the AE system was set at 35 dB; any event with a lower amplitude would not be detected. This study focused on the AE rate (number of events per second).

## 3. Results and Discussion

### 3.1. CTE Evolution

CTE fields were measured after different numbers of thermal cycles and for the three maximum cycle temperatures to assess the effect of thermal cycling on the material. For each condition, three clutch facings were tested. The following damage variable was defined to quantify the effect of thermal cycling on CTE:

(6) $$D_{i}=\frac{CTE_{i}\left(N\right)-CTE_{i}\left(0\right)}{CTE_{i}\left(0\right)}$$

where *i* = *R* or *T* and *N* is the number of thermal cycles.

**Figure 10:**
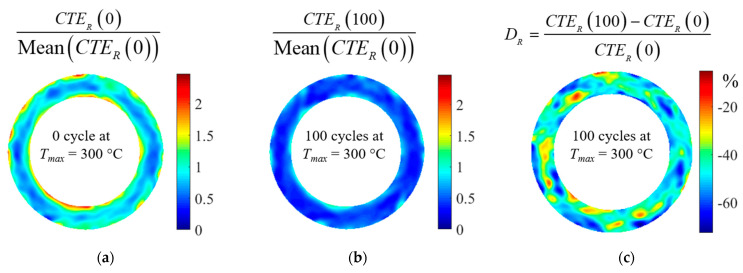
Maps of CTE after different cycles at *T_max_* = 300 °C for the same clutch facing: (**a**) initial map of *CTE_R_*, (**b**) map of *CTE_R_* after 100 cycles at *T_max_* = 300 °C and (**c**) map of *D_R_* after 100 cycles at *T_max_* = 300 °C.

The quantity *D_R_* and *D_T_* were then calculated on the disc surface as the difference between the CTE field after *N* cycles and the initial field, as shown in Figure 10 after 100 cycles at *T_max_* = 300 °C.

**Figure 11:**
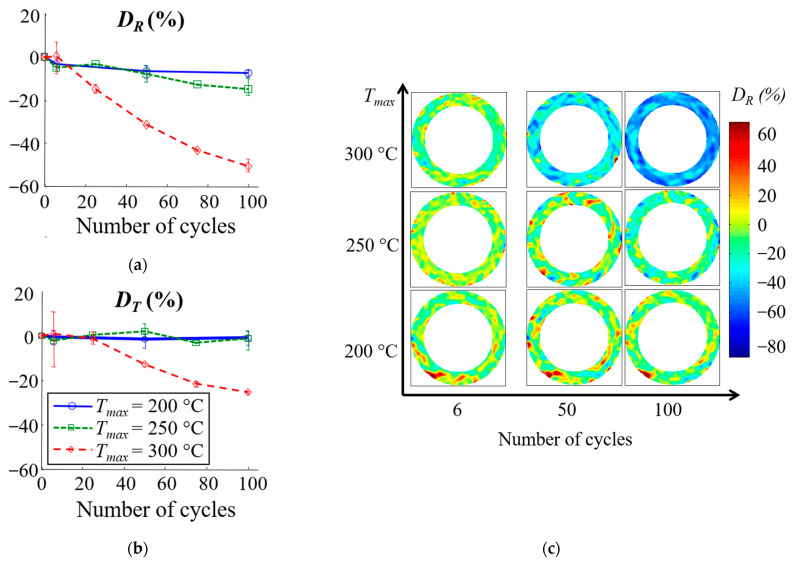
(**a**,**b**): Average evolution of *D_R_* and *D_T_* (error bars represent maximum and minimum change over three tests); (**c**) Local evolution of *D_R_* for each maximal temperature after 6, 50 and 100 cycles.

The evolutions of *D_R_* and *D_T_* as a function of the number of thermal cycles are shown in Figure 11a,b for three maximum temperature levels (200, 250 and 300 °C). As the number of cycles increases, the material shows a reduced expansion compared to its initial state. The extent of this evolution is also influenced by the maximum temperature of the cycles: the reduction in *CTE_R_* is approximately 50% for the 300 °C cycles, 15% for the 250 °C cycles and less than 6% for the 200 °C cycles. The thermal cycling has a greater effect on *CTE_R_* than on *CTE_T_*. *CTE_T_* seems not to be affected by the thermal cycles for a maximum temperature below 250 °C, whereas it decreases by about 25% for the 300 °C cycles. The results also show that for cycles at 300 °C, *CTE_T_* and *CTE_R_* converge to the same value. Indeed, initially on the material in the received state, the *CTE_R_* is twice as large as the *CTE_T_* (*CTE_T_* (0) ≈ *CTE_R_* (0)/2), and after 100 cycles at 300 °C, the *CTE_R_* decreases by about 50%, while the *CTE_T_* decreases by just over 20%. Thus, after 100 cycles at 300 °C, the radial and tangential CTE converge to the same value.

The strain fields and thus the local information on the evolution of the CTE were accessible through the experimental setup. The inhomogeneous evolution of the CTE with thermal cycling was revealed by detailed analysis of the strain fields. Figure 11c shows the evolution of the CTE fields as a function of the number of cycles for three specimens subjected to 200 °C, 250 °C and 300 °C cycles, respectively (each temperature level corresponds to one clutch facing). In some areas, the CTE increased, while in others, it decreased. For a same sample, this evolution is heterogeneous, but the distribution remains the same over cycles. The results also show that although 200 or 250 °C cycles appear to have little effect on the average material behaviour at a clutch face scale, local variations in CTE of up to 60% are possible.

**Figure 12:**
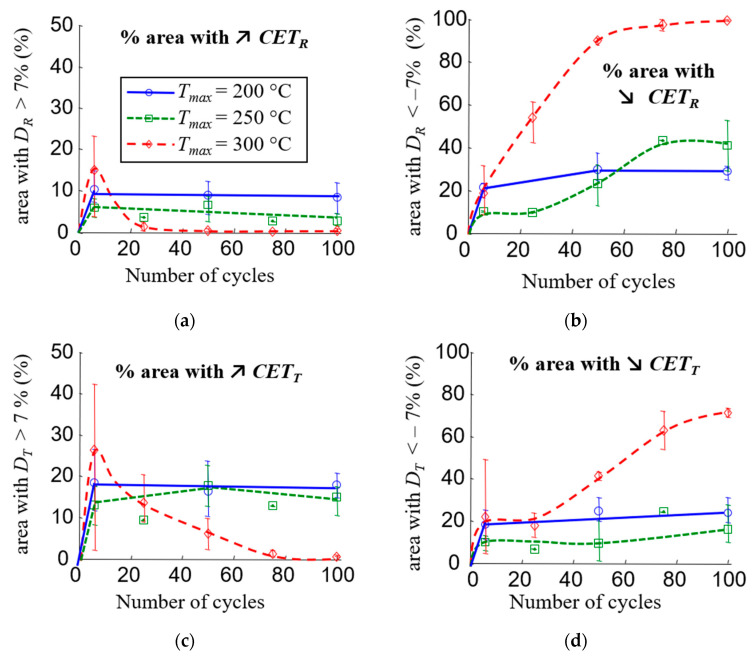
(**a**,**b**) Areas with increasing and decreasing *CTE_R_*; (**c**,**d**) areas with increasing and decreasing *CTE_T_.*

In order to quantify the areas affected by thermal cycles, a threshold depending on the strain resolution is set, and the CTE is considered modified if |*D_i_*| ≥ 7%. Figure 12 shows the areas affected by changes in CTE for the three maximum temperatures. As expected, there were more areas where the CTE decreased than where the CTE increased. This phenomenon was more significant for the *CTE_R_*. This analysis also reveals a new result: the radial and tangential CTE increased significantly in large areas (representing 8 to 30% of the total area), especially during the first cycles of thermal loading. In the case of cycles at 200 °C or 250 °C, the area of these zones quickly reached a plateau, whereas it decreased when the material was subjected to cycles at 300 °C. Regarding the *CTE_T_*, for cycles at 200 °C or 250 °C, the size of the zones where the *CTE_T_* increased or decreased is of the same order of magnitude. This explains why thermal cycling had no effect on the average *CTE_T_* for temperatures below 250 °C (Figure 11b).

Local increases and decreases in the CTE can indicate competing changes and degradation mechanisms during the thermal cycling of the material:

- the difference in the thermal expansion of the fibres and the matrix can lead to crack initiation;
- thermo-chemical reactions in the matrix can lead to the changes in the mechanical properties of the matrix but also to cracking.

In general, the presence of cracks or microvoids causes a reduction in the averaged value of the CTE [112]. However, the effect of the cracks or microvoids depends on their shape, orientation, angle with the fibres and density but also on the fibre orientation, the properties of the reinforcement and the matrix [112,113,114,115]. In this case, the CTE of the fibres is about two times lower than the CTE of the matrix (Table 1), and the manufacturing temperature is about 250 °C. Thus, the residual thermal stresses developed during manufacture place the fibre in compression and the matrix in tension along the fibre axis and inversely in the perpendicular direction. Differences in CTE between the different elements of the yarn and the matrix lead to cyclic thermal shear stress at the matrix/fibre interface, which in turn leads to decohesion and cracking. Cracks along the fibre axis make the thermal expansion of the matrix free and could locally increase the CTE of the material as long as the resin is not degraded.

The inhomogeneity of the CTE evolution at the surface of the material would be related to the heterogeneous distribution of fibres. Indeed, their orientation and density depend on the radius of the clutch facing (Figure 2b,c). Three-dimensional yarn tracing during the preforming process determines the behaviour of the clutch facing. However, certain deformations that occur during the manufacturing process can lead to irregularities, which affect the overall yarn tracing. Previous work has shown that this can have an effect on the CTE field [8]. These irregularities can therefore influence the local evolution of the CTE.

Resin ageing may also explain the average and local evolution of CTE, but further analyses were carried out to highlight this. The results are presented in the following section to help understand these phenomena.

### 3.2. Root Causes of the CTE Evolution

A non-homogeneous evolution of the material behaviour was revealed by thermal expansion measurements for different maximum temperatures. Further analysis was carried out to understand the causes of these effects. The aim was to analyse the effects of composite ageing and its impact on the CTE.

#### 3.2.1. Surface Cracking

**Figure 13:**
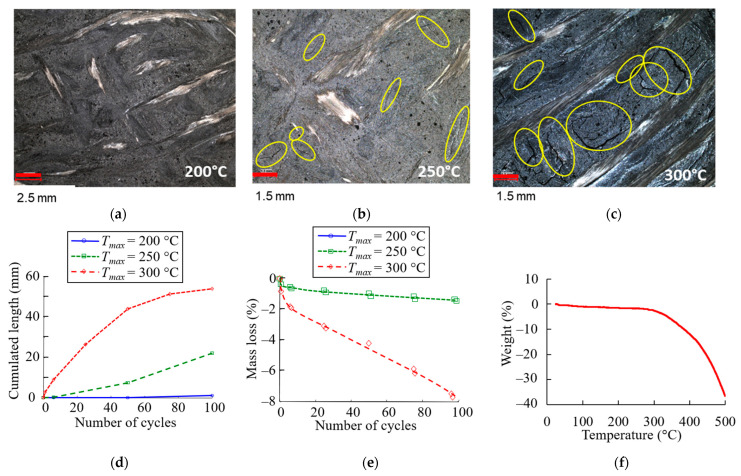
Optical observation after 100 cycles with a maximal temperature of 200 °C (**a**), 250 °C (**b**) and 300 °C (**c**) (visible surface cracks are surrounded by yellow ellipses); (**d**) Cumulated crack length; (**e**) Mass loss versus the number of cycles; (**f**) TGA results obtained on a received clutch facing in air.

First, the crack process was investigated by optical measurement (see Section 2.3.1). The number and length of cracks were measured on 12 × 12 mm^2^ surfaces (Figure 13a–c), and Figure 13d shows their evolution as a function of the number of thermal cycles.

No cracks were observed in the sample that was cycled at temperatures up to 200 °C (Figure 13a). After 100 cycles, the cumulative crack length reached 50 mm for the specimen cycled at 300 °C. This was twice that of the sample cycled at 250 °C. Cracks developed mainly along the interface between the fibre bundle and the matrix, as shown in Figure 13b,c. Crack coalescence can be observed after cycles at up to 300 °C (Figure 13c). As mentioned previously, the difference in the CTE between the glass fibre (approximately 5 × 10^−6^ K^−1^) and the thermosetting resin-based matrix (approximately 120 × 10^−6^ K^−1^; Table 1) can lead to thermal stress in the composite, which can cause matrix cracking and weakening at the fibre/matrix interface as a result of thermal cycling.

This analysis confirmed the presence of cracks in the material induced by thermal cycling, which can affect the CTE. As mentioned in the introduction, this is not the only phenomenon involved in thermal ageing. Changes to the matrix (loss of volatile substances, shrinkage due to chemical processes) are also factors to be taken into account.

#### 3.2.2. Weight Loss and DMA Analysis

A study of the mass loss of the aged materials was carried out (see Section 2.3.1) and is shown in Figure 13e. After 100 cycles, the mass loss was less 1% for 250 °C cycles, while for 300 °C cycles, the mass loss reached 8%.

Thermogravimetric analysis (TGA) performed on the clutch facing between 30 °C and 500 °C in air showed that there was no apparent weight loss below 300 °C (approx. 2.2%), at which temperature a sharp decrease is observed (Figure 13f). Various studies have shown that phenolic resins exhibit a relatively high resistance to thermal ageing [81]. The degradation of phenolic resins remains low (1–2 %) up to about 300 °C. A small percent of volatile species are emitted, consisting mainly of entrapped unreacted monomers (phenol and formaldehyde) and water, which is a by-product of further condensation reaction. It is at around 300 °C that the rate of degradation reaction increases, with the release of volatiles such as CO CO_2_, and H_2_ in addition to the previous compounds.

The TGA results and literature studies explain the difference in weight loss observed between aged samples. The sharp increase in weight loss during thermal fatigue at 300 °C is related to the coupling between oxidation and repeated thermal loading.

**Figure 14:**
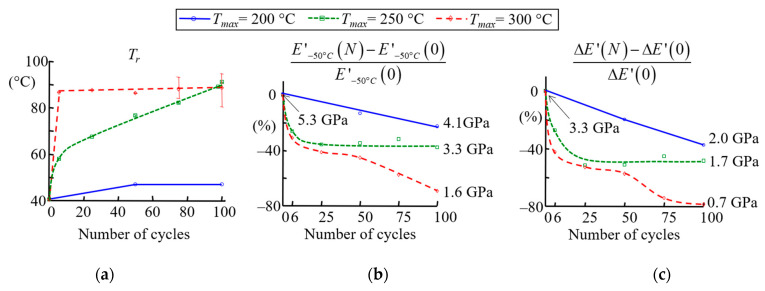
Evolution of *T_r_* (**a**), storage modulus *E*′ (for T=−50°C) (**c**) and ΔE′ (**b**).

In order to analyse the thermal ageing of the matrix, Dynamic Mechanical Analysis (DMA) tests were carried out (see Section 2.3.2) on materials after different ageing treatments (number of cycles and load temperature *T_max_*). Figure 14 shows the evolution of the relaxation temperature T_r_, the storage modulus at −50 °C *E*′_−50°C_ and the modulus difference Δ*E*′= *E*′_−50°C_ − *E*′_150°C_ as a function of the number of cycles and for three load temperatures.

When the specimen is cycled at 200 °C, T_r_ and *E*′_−50°C_ are slightly affected compared to those at load temperatures of 250 or 300 °C. At cycling temperatures above 200 °C, *T_r_* increases with the number of cycles and tends to plateau (Figure 14a). The formation of additional cross-links with thermal ageing due to reactions of unreacted monomers may be responsible for this increase. This is confirmed by the decrease in Δ*E*′ with the number of thermal cycles (Figure 14c). In addition to post-curing [51], the increase may be linked to oxidative crosslinking, as cited previously. The post-crosslinking processes under heating in the presence of oxygen have already been demonstrated on diene elastomers used in brake pad formulations [107]. Other authors have shown that degradation phenomena are accelerated by the presence of heavy metals, such as copper, for these types of elastomer [116]. However, *E*′_−50°C_ decreases when samples are subjected to 250 °C or 300 °C cycling (Figure 14b). As mentioned above, this could be due to the presence of cracks that form during thermal cycling. The ageing kinetics are much greater at 300 °C than at 250 °C, where the material seems to reach a stabilised state (constant values of *E*′_−50°C_ and Δ*E*′). Damage at 300 °C appears to be fast and very significant, with the storage modulus decreasing very drastically.

In conclusion, resin transformation and, probably, additional cross-linking due to the thermal ageing of the matrix can be observed. This modification of the resin could contribute to a reduction in the CTE of the material due to the molecular mobility limited by additional cross-linking. The inhomogeneity of the CTE evolution at the surface of the material would then be related to a heterogeneous evolution of the resin but also to the fibre distribution and orientation. The ageing of the mixture could be accelerated by the copper. The degradation is certainly not homogeneous in depth and on the surface. Furthermore, crack initiation can have two origins: (i) the difference in the thermal expansion of the glass fibre and the matrix; (ii) the thermo-chemical reaction in the matrix. Cracking and the evolution of CTE were observed on the surface of clutch facings, but the effects of thermal cycling within the thickness have not been quantified. The influence of the thermal cycling within the volume is presented in the next section using tensile tests coupled with acoustic emission measurements. The latter allowed us to highlight the presence of damage caused by thermal cycling, as well as changes in mechanical properties.

#### 3.2.3. Highlighting of Volume Damage: Ageing, Cracking and Mechanical Properties

The analyses presented so far (CTE evolution, DMA, mass loss and surface cracking) suggest that the material evolves little when cycled at 200 °C and much more when cycled at 300 °C. To verify this, tensile tests coupled with acoustic emission (see Section 2.3.3) were carried out to provide indirect evidence of the volume evolution of the material. This also complemented the analysis of the crack observation, which was only carried out on the surface. Three types of specimens were considered: as-received, after six cycles at 200 °C and after six cycles at 300 °C. For each type, three specimens were tested under monotonic tensile tests, and the results are reproducible. In the following, only information relevant to the understanding of the observed phenomena is exposed. The interested reader can find more information in [108].

**Figure 15:**
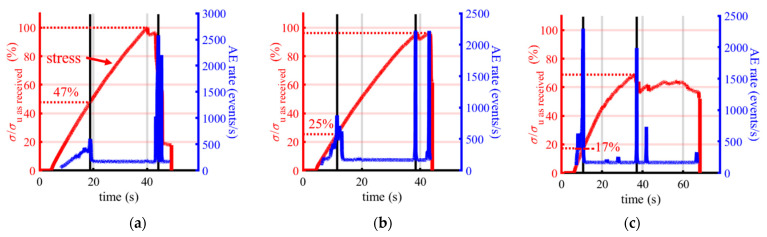
Stress and AE rate versus time for: (**a**) as-received specimen; (**b**) after six 200 °C cycles and (**c**) after six 300 °C cycles.

For the three states, the time evolutions of the stress-to-the-ultimate strength ratio and the AE emission rate are shown in Figure 15.

Figure 15a shows the results obtained on an as-received specimen, and it is possible to identify three phases from the evolution of the acoustic emission (AE) rate as a function of time. The first phase, during which the acoustic emission rate (AE) increases significantly, indicates uncontrolled defect growth [117]. This phase ends when the load reaches 47% of the maximum stress. The second phase, in which the acoustic emission rate suddenly decreases and stabilises at around 100 events per second, indicates controlled defect growth [117]. In the third phase, the AE emission rate reaches a peak for the second time, and failure of the specimen occurs. Figure 15b,c show the AE signals for the two specimens subjected to six thermal cycles at 200 °C and 300 °C, respectively. The three phases were identified using the same criteria based on the AE rate. The results show that the higher the stress temperature, the more the boundary between the first two phases occurs at lower stress levels. Indeed, in the case of cycling at 200 °C, while stress evolution appears to be unaffected by thermal cycling, phase change occurs at a stress equivalent to 25% of the maximum stress of the as-received material. For cycling at 300 °C, this phase change occurs at 17% of this maximum stress. These results indicate the presence of pre-existing damage prior to the tensile tests, which is due to the thermal cycling and which increases with the load temperature. Even samples cycled at 200 °C are affected by thermal cycling and show signs of damage after a small number of cycles. The use of AE signals as a damage monitoring technique therefore provides additional information on degradation in the volume, complementing the results discussed in the previous sections.

**Figure 16:**
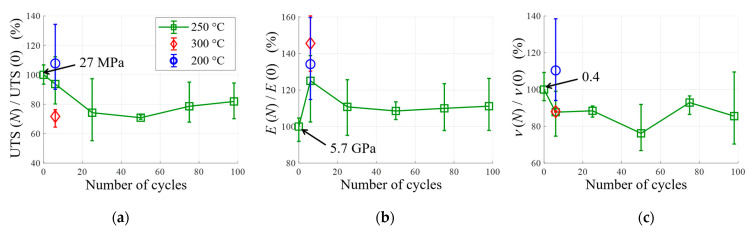
Evolution of UTS (**a**), Young’s modulus *E* (**b**) and Poisson’s ratio *ν* (**c**).

To quantify the effect of thermal cycling on the material’s mechanical properties, additional tensile tests were carried out. Figure 16a shows the evolution of the Ultimate Tensile Strength (UTS) as a function of the number of cycles at 250 °C and after six cycles at 200 °C or 300 °C. The UTS is defined as the maximum stress reached during the tensile test, and the error bars represent the maximum and minimum UTS for three specimens. These measurements were made difficult by the fact that the rectangular samples were not flat after thermal cycling. Indeed, as the thermal cycling was performed without mechanical stress, the samples were twisted (internal stress release, cracking). For this reason, the test campaign was not complete. Considering the scatter of the results, thermal cycling seems to have little effect on the mechanical strength of the specimen cycled at 200 °C. The UTS decreases slightly by about 20–30% after loading at 250 °C. However, after six cycles at 300 °C, the thermal cycling had a greater effect on mechanical strength. The significant damage above 300 °C is therefore confirmed by these tensile tests. However, it is important to note that the imposed thermal cycling is particularly severe, as heating is uniform throughout the part. In reality, the heating is limited in the thickness of the material due to its insulating nature and the time over which it is subjected to the temperature increase.

Figure 16b,c show the evolutions of the elastic properties of the material (Young’s Module and Poisson’s ratio) as a function of the number of thermal cycles. These results seem to confirm those obtained with DMA. After six cycles, the higher the temperature, the greater the increase in stiffness. When the material is cycled at 250 °C, the elastic properties quickly reach asymptotic values. This increase in Young’s modulus is in line with the observed decrease in CTE.

The results presented in this section therefore confirm the analyses and hypotheses made previously: thermal cycling leads to an evolution of the matrix and volume damage, even at the lowest temperatures. This ageing leads to changes in mechanical properties, i.e., a reduction in the CTE and ultimate tensile strength, plus an increase in Young’s modulus.

#### 3.2.4. Summary

Three mechanisms have been identified that may explain the change in the coefficient of thermal expansion. The first is the cracking of the matrix associated with the difference in the coefficient of expansion, which is the only one that can explain the local increase in CTE. The other two are subjected to the ageing of the matrix: cracking induced by its retraction and a change in mechanical properties induced by additional cross-linking. The results of Figure 11c and Figure 12 seem to indicate that the first mechanism occurs rapidly, after only a few cycles, and that the evolution of the thermal expansion coefficient is then linked to the other two. At 200 °C, the first mechanism appears to be predominant, whereas at 300 °C, the ageing of the matrix is rapidly imposed, which explains this large drop in the coefficient of thermal expansion. At 250 °C, ageing is more gradual, which implies this intermediate evolution. Finally, the heterogeneous evolution of the CTE seems to be related to the fibre distribution and orientation. The ageing of the resin could be accelerated by the copper, and the cracking process depends directly on the mechanical interaction between the matrix and fibre.

## 4. Conclusions

The objectives of this study were twofold: first, to evaluate the effects of thermal cycling on the coefficients of the thermal expansion distribution of a clutch facing using an original experimental technique and, second, to analyse the results by characterising the effects of ageing on the material. The results show that: the greater the number or the maximal temperature of cycles is, the greater the drop in the averaged coefficients of thermal expansion. Regarding the thermal expansion coefficient fields, some areas show an increase while others show a decrease. Additional analyses were carried out in order to understand these results. On the one hand, the presence of cracks in the matrix caused by differential expansion can be observed. On the other hand, resin transformation and, probably, additional cross-linking due to the thermal ageing of the matrix induce further cracks and the evolution of thermo-mechanical properties. The interaction of these complex thermal degradation mechanisms between them and the fibres leads to a non-homogenous evolution of the CTE. The actual fibre organisation is very heterogeneous. Ageing seems to be activated at a temperature between 200 and 250 °C. At 200 °C, only the cracking induced by the coefficient of thermal expansion differences seems to occur. At 250 °C, the ultimate tensile strength of the material is reduced by 20–30% after 100 cycles. Finally, it is important to stress the fact that the imposed thermal cycling is very severe due to the homogeneous heating of the part. In reality, the clutch facing is submitted to a high but very short increase in the temperature and only on the sliding surface. The next steps in this work would be to develop a constitutive model of the thermal fatigue of this material and to perform tests where only the surface of the clutch is heated.

## Figures and Tables

**Table 1:** Mechanical properties of the main constituent materials used in the composite.

Material	CTE (×10^−6^ K^−1^)	Young’s Modulus *E* (GPa)	Ultimate Tensile Strength (MPa)
PAN	80–90	17–19	200–420
E glass fibre	4.9–5.1	73	2000–2500
Copper fibre	17	122–128	300–400
Phenolic/Melamine	120–125	2–4	30–60
SBR + Carbon Black	160–180	0.004–0.006	16–26

## Data Availability

Not applicable.

## Abbreviations

The following abbreviations are used in this manuscript.

α	Coefficient of the thermal expansion tensor
Δ*T*	Temperature variation
Δ*E*′	Drop in storage modulus
ɛ	Strain
ɛ_T_, ɛ_R_	Tangential and radial strains
*ν*	Poisson’s ratio
θ	Polar angle in the polar coordinate system
*A*_0_	Track width (clutch facing)
AE	Acoustic Emission
BSE	Backscattered Electron Detection
CTE	Coefficients of thermal expansion
*CTE_R_*, *CTE_T_*	Radial and tangential coefficients of thermal expansion
*D_i_*	Damage variable
*D_R_, D_T_*	Radial and tangential damage variable
DIC	Digital image correlation
DISC	Digital Image Stereo-Correlation
DMA	Dynamical mechanical analysis
*D_out_*, *D_in_*	Outer and inner diameters (clutch facing)
*EDS*	Energy Dispersive Spectroscopy
*E**	Complex Young modulus
*E*′	Storage Young Modulus
*E″*	Loss Young Modulus
*L_b_*	Preforming ratio (clutch facing)
*N*	Number of cycles
*NBR*	Nitrile Butadiene Rubber
*R*_0_	Mean radius of the clutch facing
ROI	Region of interest
SBR	Styrene Butadiene Rubber
SEM	*Scanning Electron Microscope*
tan *δ*	Loss modulus
*T_g_*	Glass transition temperature
Ti	Relaxation temperature
TGA	Thermogravimetric analysis
UTS	Ultimate Tensile Strength
*U_r_, U_t_*	Radial and tangential strain directions
